# Pharmacological and Non-Pharmacological Augmentation of Clozapine Treatment in Psychosis in Children and Adolescents: A Systematic Review

**DOI:** 10.1192/bjo.2026.11174

**Published:** 2026-06-30

**Authors:** Umraj Rai, Kiranjeet Gill, Ramya Srinivasan

**Affiliations:** University College London Hospitals, London, United Kingdom

## Abstract

**Aims:**

Children and adolescents with psychosis are more likely to have a poor prognosis and to experience treatment resistance, than adults. This is associated with a reduced quality of life, as well as increased care needs and economic cost. Clozapine is recommended for treatment-resistant psychosis because of greater efficacy than other antipsychotics, however this can be insufficient. Clozapine can be augmented with a range of different treatments, however there is relatively little data available for this in children and adolescents.

**Methods:**

A systematic search was carried out of four databases (Embase, Medline, PsycInfo, CINAHL) from inception to February 2026, to identify studies of pharmacological and non-pharmacological clozapine augmentation strategies in children and adolescents, including psychotherapeutic or psychosocial interventions, neuromodulation, and nutritional or dietary supplements. To be eligible for inclusion, patients in the study needed to be prescribed clozapine prior to initiation of the adjunctive treatment, and to have outcome measures recorded before and after augmentation. The study was registered on PROSPERO (CRD42024564242).

**Results:**

Nine studies were found that reported on children or adolescents (n=59) receiving medications, ECT or other neuromodulation techniques to augment clozapine. The majority (6/9) were case reports or case series, retrospective chart reviews and one pilot RCT. Some clinical improvement was reported for a majority of patients in the included studies, however this was mostly short-term. No reports were found on adjunctive psychotherapeutic or psychosocial intervention, nor nutritional or dietary supplementation.

**Conclusion:**

Some clozapine augmentation strategies may be effective in children and adolescents, but available evidence is limited and very weak. Of note, the search identified a number of studies which could not be included due to lack of outcome measures. This highlights the potential importance of routinely collected outcome data, in building an evidence base for rare presentations. Research into psychotherapeutic or psychosocial treatments was also lacking. Further high-quality, prospective research is needed to address this gap.

